# Differential Expression of ZNF83, ACY-1, and SMC5 in Cumulus Cells: A Potential Biomarker for Oocyte Fertilization Competence

**DOI:** 10.5935/1518-0557.20260022

**Published:** 2026

**Authors:** Azam Govahi, Ehsan Raoufi, Mahsaneh Vatankhah, Elham Hosseini, Hadis Gholipour, Fatemehsadat Amjadi

**Affiliations:** 1 Endometriosis Research Center, Iran University of Medical Sciences, Tehran, Iran; 2 Vaccina Research Center, Iran University of Medical Sciences, Tehran, Iran; 3 Department of Anatomy, School of Medicine, Iran University of Medical Sciences, Tehran, Iran; 4 Social Determinants of Health Research Center, Health and Metabolic Diseases Research Institute, Zanjan University of Medical Sciences, Zanjan, Iran; 5 Department of Obstetrics and Gynecology, Mousavi Hospital, School of Medicine, Zanjan University of Medical Sciences, Zanjan, Iran; 6 Infertility and IVF Department, Firouzabadi Educational and Medical Center and Hospital, Iran University of Medical Sciences, Tehran, Iran; 7 Reproductive Sciences and Technology Research Center, Department of Anatomy, Iran University of Medical Sciences, Tehran, Iran

**Keywords:** cumulus cells, ZNF83, ACY-1, SMC5, oocyte quality

## Abstract

**Objective:**

Since cumulus cells (CCs) play an undeniable role in oocyte maturation by producing and transferring important molecules to the oocyte, examining these cells can provide a broad view of the factors affecting oocyte competence. Therefore, the aim of this study was to investigate the expression of genes and proteins *ZNF83, ACY-1*, and*SMC5* in CCs of unfertilized oocytes and compare it with fertilized oocytes to gain new insights into the relationship between CCs function and oocyte competence.

**Methods:**

Eighteen healthy female oocyte donors were included in this study. After obtaining the cumulus-oocyte complex and isolating the CCs, oocytes were injected and the embryos were followed and morphologically graded on day 3. Then the expression of genes and proteins *ZNF83, ACY-1*, and*SMC5* in CCs of non-fertilized oocytes and CCs from fertilized oocytes was investigated and compared.

**Results:**

The expression of *ZNF83, ACY-1*, and *SMC5* at gene and protein levels was reduced in the non-fertilized group compared to the fertilized group (*p*<0.05).

**Conclusions:**

The results highlight CCs’ critical role in supporting oocyte fertilization. These findings show the way for novel biomarkers and therapeutic strategies to improve IVF outcomes. More functional studies are needed to support the hypothesis of this study and explore the clinical applications of these markers in ART.

## INTRODUCTION

Oocyte quality plays an important role in the success of assisted reproductive technology (ART) procedures (Li *et al.*, 2008; Assou *et al.*, 2010). Communication between the oocyte and the surrounding cumulus cells (CCs) via gap junctions and paracrine signals is essential for oocyte competence and influences fertilization outcomes (Molinari *et al.*, 2016).

CCs support oocyte nuclear and cytoplasmic maturation by transferring required molecules for oocytes such as amino acids and protecting the oocytes against harmful factors such as oxidative stress (Eppig *et al.*, 2005; Uhde *et al.*, 2018; Martinez *et al.*, 2023). They also sustain energy within the cumulus-oocyte complex (COC) by providing required molecules for ATP synthesis, like pyruvate, lactate, and cholesterol precursors (Da Broi *et al.*, 2018). On the other hand, oocytes regulate CCs differentiation, proliferation and apoptosis through the secretion of paracrine factors (Hourvitz *et al.*, 2010; Huang & Wells, 2010). Any disruption of this cross-talk might impair the oocyte competence and fertilization outcomes (Da Broi *et al.*, 2018). Despite technological progress, molecular markers of oocyte competence within CCs are not completely understood. Recent studies have suggested that CC-derived transcription factors (TFs) are linked to embryo quality (Wathlet *et al.*, 2011; Burnik Papler *et al.*, 2015; Li *et al.*, 2015a; 2015b; Shen *et al.*, 2020).

However, the specific role of TFs in CCs performance is still poorly characterized. TFs such as Zinc Finger 83 (ZNF83), and related genes including structural maintenance of chromosomes 5 (SMC5), and Aminoacylase 1 (ACY-1) have been suggested as candidates for CCs performance based on their involvement in critical pathways. For example ZNF83 regulates RNA polymerase II activity, potentially modulating RNA supply to the oocyte (Lambert *et al.*, 2018); while animal studies have shown that SMC5 maintains chromosomal stability during meiosis (Hwang *et al.*, 2017; Pradhan *et al.*, 2023); and ACY-1 facilitates amino acid recycling and oxidative stress defense (Al-Helaly & Mahmood, 2021; Fernández-Hernández *et al.*, 2021). These genes selected based on transcriptomic data from a prior screen (Govahi *et al.*, 2022) and it seems to be effective on the function of CCs and the two-waycommunication between CCs and oocytes by affecting processes such as cell division, DNA damage repair (Pradhan *et al.*, 2023), protection against oxidative stress (Al-Helaly & Mahmood, 2021), and providing the oocyte with the necessary materials (Fernández-Hernández *et al.*, 2021).

We hypothesized that dysregulation of these TFs in CCs would correlate with oocyte fertilization failure. Our study aims to compare the expression of *ZNF83, ACY-1*, and *SMC5* in CCs by fertilized oocytes producing high-quality embryos versus non-fertilized oocytes following intracytoplasmic sperm injection (ICSI).

## MATERIALS AND METHODS

### Sample Collection

The study began after approval by the Ethics Committee of Iran University of Medical Sciences (Approval ID: IR.IUMS.REC.1400.509) and obtaining informed consent from all participants. Eighteen oocyte donors meeting the following inclusion criteria were enrolled:

- Proven fertility (≥1 live birth from natural conception)
- No reproductive tract abnormalities or underlying diseases
- Age <35 years and BMI 18-28
- The partner of the couples receiving the donated oocyte was aged <45 years

To minimize confounding effects of paternal factors on fertilization and embryo quality, sperm samples with abnormal semen parameters (outside WHO standards) or DNA Fragmentation Index (DFI) >20% were excluded (Kandil *et al.*, 2021). Antagonist protocol was used for stimulation. Cumulus-oocyte complexes (COCs) were collected via transvaginal ultrasound. After a 2-hour incubation, CCs were mechanically denuded from Metaphase II (MII) oocytes. MII oocytes were placed in labeled culture droplets for intracytoplasmic sperm injection (ICSI). Injected oocytes were maintained in G1 medium until day 3 of development. CCs from each oocyte were centrifuged and cryopreserved in liquid nitrogen (-196°C). Two samples were taken from everyone. CC from fertilized oocyte that formed high-quality embryos (6-8 cell, no fragmentation, evenly sized blastomeres) (Sakkas & Gardner, 2017) were considered as the fertilized group and CC from oocyte failing to fertilize were considered as the non-fertilized group.

### Sperm DNA Fragmentation

Sperm DNA fragmentation (SDF) assay was performed by a DNA fragmentation assay kit (Dianbio Assay Co.). Assessment was done according to the manufacturer’s protocol. Briefly, dilution of semen to achieve concentration of 5-10 × 10^6^ sperm/mL was performed by phosphate-buffered saline (PBS; pH 7.4). Diluted semen was embedded in low-melting-point agarose gel. Subsequently, samples mounted on pre-coated slides. Cellular denaturation and lysis were performed using acid and lysis solutions, respectively, followed by dehydration through a graded ethanol series (70%, 90%, and 100%). Two hundred sperms per sample were analyzed, with DNA fragmentation classified based on the presence or absence of characteristic halo patterns: spermatozoa exhibiting minimal or no halos were classified as containing fragmented DNA. DFI was calculated as the percentage of spermatozoa with abnormal halo formation. In alignment with clinical thresholds, a DFI value ≤ 20% was deemed within the normal range (Mirsanei *et al.*, 2023).

### Gene expression assessment by RT-qPCR

Total RNA extraction was performed using the Trizol reagent (Sigma, Poole, UK). Spectrophotometry (NanoDrop) was used for RNA concentration and purity assessment. Then, Complementary DNA (cDNA) was synthesized by reverse Transcription Kit (Bio-Rad, #1725037), according to the manufacturer’s instructions. Quantitative PCR (qPCR) was conducted in triplicate to evaluate the expression levels of target genes (*ZNF83, ACY-1*, and*SMC5*). The relative gene expression was calculated using the 2-∆∆Ct method. Primer sequences used in the assay are listed in Table 1.

**Table 1:** Primer sequences used in the assay

Gene	Sequence (5-3)	Length	Gene ID
*ZNF83*	Forward: GTAATGAATGTGGGAAGGT	19	55769
	Reverse: TGATGATTAGTGAGGCTTG	19	
*ACY-1*	Forward: GGCTGCATGAGGCTGTGTT	19	95
	Reverse: CTTGGCACTGGTTGGGATG	19	
*SMC5*	Forward: GTCTGGTAGAACTGGTAATGTGC	23	*23137*
	Reverse: ATCTGGGGCTGAAGATTCTCG	21	
*(βactin)*	Forward: CAAGATCATTGCTCCTCCTG	20	60
	Reverse: ATCCACATCTGCTGGAAGG	19	

### Proteins expression assessment by Western Blot

We used CCs from two groups and performed Western blot analyses as previously described, with some modifications (Jabarpour *et al.*, 2018; Gholipour *et al.*, 2023). Briefly, the cells were lysed with ripa buffer. The lysates were removed by centrifugation. Protein concentration was determined by the Bradford Protein Quantification kit (DNAbioTech, Iran). The tissue lysates were mixed with a Laemmlisample buffer. Lysates were then subjected to SDS-PAGE and subsequently transferred to a 0.2 µm Immune-Blot™ polyvinylidene difluoride (PVDF) membrane (Bio-Rad Laboratories, CA, USA). The membranes were then blocked with 5% BSA (Sigma Aldrich) in 0.1% Tween 20. Then, the membranes were incubated with Anti-ACY-1 (abcam), Anti-SMC5 (abcam), Anti-ZNF83 (abcam), and anti-β actin-loading control antibodies (Abcam). After washing and incubation of the membranes, goat anti-rabbit IgG H&L (HRP) (Abcam) secondary antibody was used. The membranes were then incubated with enhanced chemiluminescence (ECL). β-actin was used for normalization.

### Statistical analysis

Data was presented as mean ± standard deviation (SD). Statistical comparisons were performed using independent sample-test. All analyses were conducted using**GraphPad Prism version 8.0**, with *p***-value <0.05** considered statistically significant.

## RESULTS

### RT-qPCR Analysis

**Figure 1:**
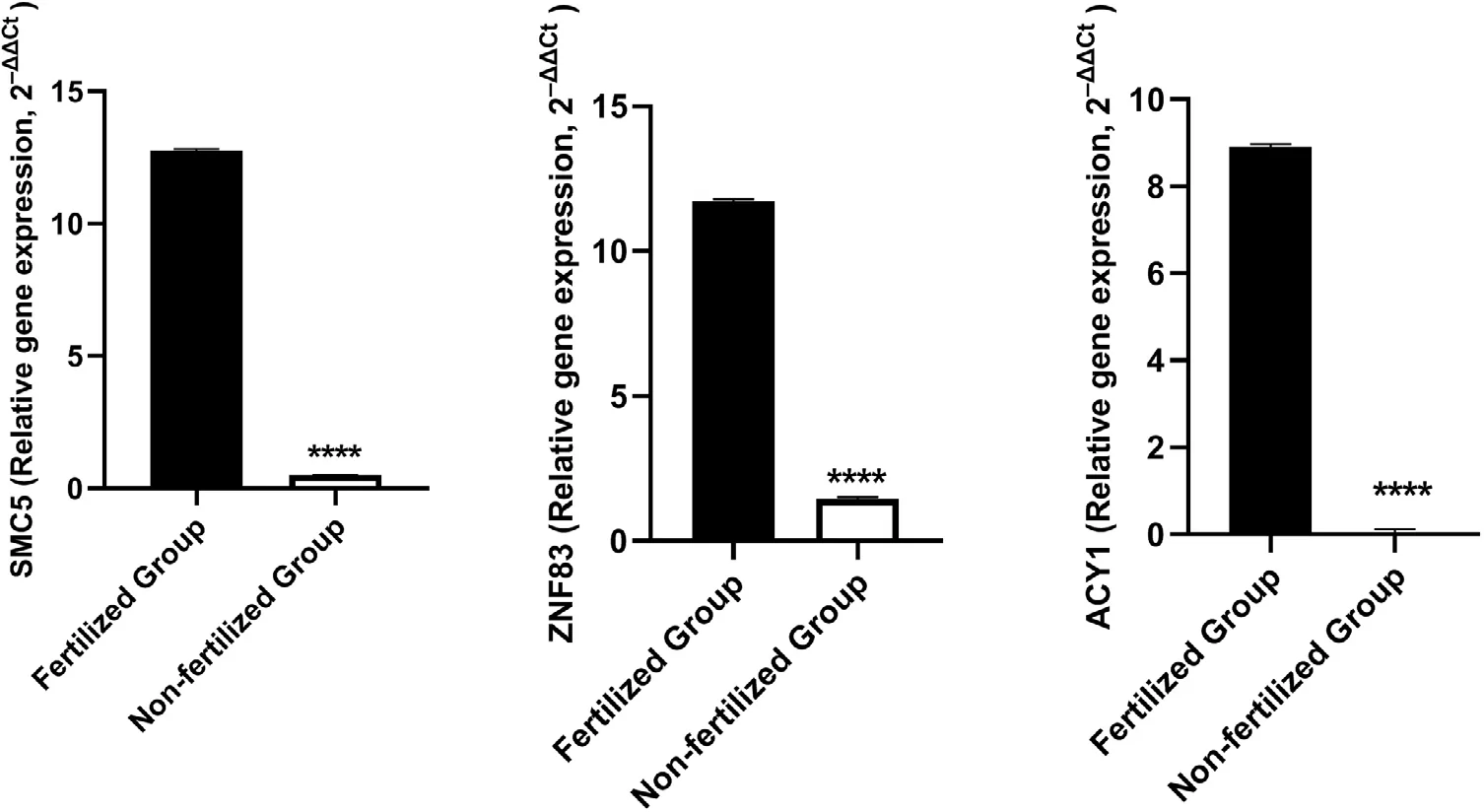
Real-time PCR analysis of ZNF83 (zinc Finger 83), ACY-1 (aminoacylase-1), and SMC5 (structural maintenance of chromosomes 5) genes expression in non-fertilized groups versus fertilized group. Expression level of all three genes (ACY-1, SMC5, ZNF83) were significantly downregulated in the non-fertilized group compared to the fertilized group (*p*<0.0001****). β-actin was used for normalization. Number of biological replicates (n): 3.

As described in Figure 1, Real-time results showed that the expression of*ZNF83* was significantly downregulated in the non-fertilized group than in the fertilized group (*p*<0.0001). Similarly, the expression of genes *ACY-1* and*SMC5* was also significantly reduced in the non-fertilized group compared to the fertilized group (*p*<0.0001).

### Western blot Analysis

**Figure 2:**
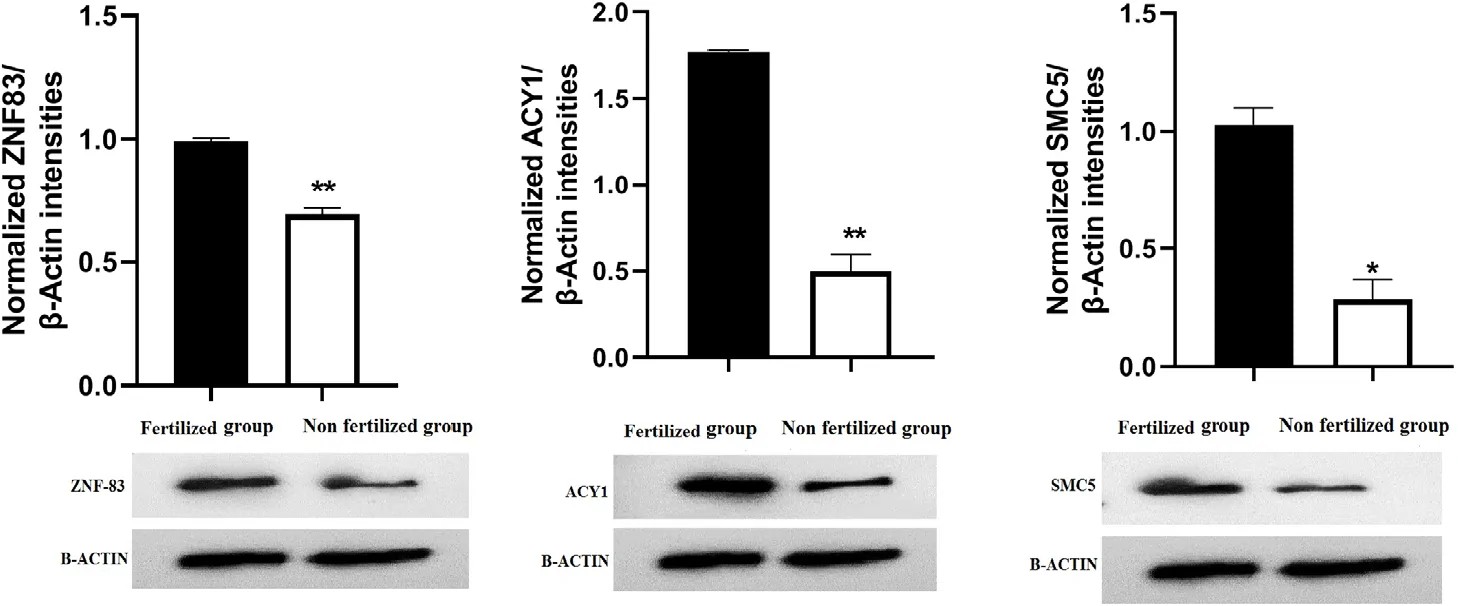
Western blot analysis ofZNF83 (zinc Finger 83), ACY-1 (aminoacylase-1), and SMC5 (structural maintenance of chromosomes 5) expression. Protein levels of three target (ACY-1, SMC5, ZNF83) showed significant downregulation in the non-fertilized group compared to the fertilized group (*p<0.05/ **p<0.01). Molecular Weight: ZNF83: 60kda, ACY-1:46kda, SMC5:129kda, β-actin:42kda. β-actin was used for normalization. Number of biological replicates (n):2.

As described in Figure 2, Western blot results revealed that the protein expression of *ZNF83* was significantly downregulated in the non-fertilized group than in the fertilized group (*p*<0.01). Similarly, the protein expression of*ACY-1* and *SMC5* was also significantly reduced in the non-fertilized group compared to the fertilized group (*p*<0.01, *p*<0.05).

## DISCUSSION

This study compared the gene and protein expression levels of *ZNF83, ACY-1*, and *SMC5* in CCs from oocytes that failed to fertilize (non-fertilized group) and CCs from oocytes that successfully fertilized and developed into high-quality embryos (fertilized group) following ICSI. A parallel decrease in both mRNA and protein expression of *ZNF83, ACY-1*, and *SMC5* in the non-fertilized group was observed in this study. These findings suggest that these molecules may play a functional role in oocyte developmental competence and could serve as novel biomarkers for predicting fertilization potential.

Due to the well-established role of CCs in oocyte maturation, fertilization, and early embryo development, functional impairments of these cells may contribute to reduced fertilization rates (Turathum *et al.*, 2021). McReynolds *et al.* (2012) demonstrated that alterations in the proteomic profile of CCs is associated with follicular senescence. Li *et al.* (2015a) have reported increased mRNA levels of PRSS35 in CCs from fertilized oocytes compared to non-fertilized oocytes. Reduced HLA-G expression in CCs has been shown to be associated with poor oocyte quality and impaired embryo development (Aftabsavad *et al.*, 2021).

Many studies have reported that, in addition to small molecules, large molecules such as long RNA are transferred from CCs to the oocyte (Macaulay *et al.*, 2014). One of the key functions of CCs is to supply the RNA required by the oocyte to provide maternal reserves (Russell *et al.*, 2016). Until embryonic genome activation, the developing embryo relies on the oocytes’ RNA for proper development (Hand *et al.*, 2017). Maternal RNA reserves in the oocyte are stored as ribonucleoprotein complexes. Since CC-derived transcripts do not require the dissociation of these protein complexes for translation, their translation within the oocyte is more efficient and accessible. These transcripts are primarily involved in critical processes such as meiosis regulation, transcription, and translation. According to (Macaulay *et al.*, 2016), this transfer follows a precise timeline, occurring just before meiosis resumption. Notably, some of these transferred transcripts increase when oocyte transcription is silenced, suggesting their functional importance for the oocyte’s function (Macaulay *et al.*, 2014; 2016).

In our study, *ZNF83* expression was significantly lower in the infertile group. Considering its role as a TF, a reduction in *ZNF83* expression among CCs may impair the synthesis of essential RNAs and their transfer to the oocyte, leading to reduced oocyte competence. Also, *ZNF83* plays a critical role in cell cycle regulation via its interaction with RNA polymerase II, so its downregulation could disrupt the cell cycle, leading to aberrant cell division and increased apoptosis rates.

We also observed that SMC5, a component of the chromosomal maintenance complex, was notably downregulated in the non-fertilized group. SMC5 plays a critical role in gene expression regulation, chromosome segregation, and DNA damage repair (Pradhan *et al.*, 2023). Consequently, defects in this protein may impair DNA repair mechanisms. If DNA damage remains unrepaired due to a compromised repair system, it often leads to apoptosis (De Zio *et al.*, 2013) and increased apoptosis in CCs can diminish their ability to support the oocyte, potentially contributing to abnormal embryonic cleavage divisions (Burrows *et al.*, 2006). Notably, gametes and embryos derived from COCs with minimal or no apoptosis exhibit a higher developmental competence, reaching the blastocyst stage more efficiently (Corn *et al.*, 2005). Additionally, research in mouse oocytes has shown that SMC5 levels decline with maternal age, and this protein is essential for the correct segregation of homologous chromosomes during meiosis I (Hwang *et al.*, 2017). Based on these functional properties of SMC5, the reduced expression of SMC5 in the CCs of the non-fertilized group could contribute to lower oocyte fertilization potential.

We also observed reduced expression of the ACY-1 gene and protein in CCs of non-fertilized oocytes compared with fertilized oocytes. This enzyme hydrolyzes acetylated proteins, leading to protein recycling into amino acids (Fernández-Hernández *et al.*, 2021). CCs supply vital amino acids to the oocyte, and their absence impairs oocyte metabolism, fertilization and early embryo development (Martinez *et al.*, 2023). Prior studies have reported that embryo cleavage divisions depend on amino acids provided by CCs. In the absence of CCs, only exogenous amino acid supplementation supports proper division (Juetten & Bavister, 1983). Thus, the observed ACY-1 downregulation in non-fertilized oocytes may impair amino acid supply, reducing the oocytes’ fertilization potential.

ACY-1 also can protect against oxidative stress by increasing glutathione peroxidase and reducing malondialdehyde (Al-Helaly & Mahmood, 2021). Elevated reactive oxygen species (ROS) in CCs is linked to follicular senescence and infertility (Lin *et al.*, 2020). Therefore, lower ACY-1 levels in non-fertilized oocytes could heighten oxidative stress, thus weakening the CCs’ ability to support oocyte fertilization and embryo development. Significantly reduced expression of ZNF83, ACY-1, and SMC5 in CCs from non-fertilized oocytes highlights their critical role in supporting oocyte fertilization. The consistency between gene and protein expression patterns reinforces their functional relevance in CC-mediated oocyte support.

Given the reproductive health of the study groups, it is not possible to say for sure what factors may have affected the quality of CCs and the reduction expression of these genes. The observed decrease in the expression of these genes in the non-fertilized group could indicate a disruption in the communication between the oocyte and the CCs. Because in addition to supporting the CCs from the oocyte, the oocyte also affects the proliferation and activity of these cells by secreting a number of factors such as GDF9/BMP15 (Su *et al.*, 2008). Therefore, one possibility could be a decrease in the signaling of the oocyte itself. Epigenetic changes (such as Histone modifications and DNA methylation) cannot be ignored either. These changes, which can be the result of intrinsic (endogenous) and extrinsic (environmental) factors, can affect the expression of the genes (Nalvarte *et al.*, 2018). Considering the limitations of direct examination of the human oocyte, designing studies with the aim of controlling clinical variables and functional assays of these genes in CCs can help to identify the responsible factors in this field”.

These findings show the way for novel biomarkers and therapeutic strategies to improve IVF outcomes. More functional studies are needed to support the hypothesis of this study and explore the clinical applications of these markers in ART.
